# Clinical phenotype of high-impact chronic pain in sickle cell disease at consultation for hematopoietic cell transplant

**DOI:** 10.1093/jscdis/yoaf028

**Published:** 2025-08-21

**Authors:** Serena Huang, Scott Gillespie, Eric Chou, Katie Liu, Ashna Jagtiani, Lakshmanan Krishnamurti, Nitya Bakshi

**Affiliations:** Emory University School of Medicine, Atlanta, GA 30322, United States; Pediatrics Biostatistics Core, Department of Pediatrics, Emory University, Atlanta, GA 30322, United States; Division of Pediatric Hematology-Oncology-BMT, Department of Pediatrics, Emory University School of Medicine, Atlanta, GA 30322, United States; Pediatrics Biostatistics Core, Department of Pediatrics, Emory University, Atlanta, GA 30322, United States; Division of Pediatric Hematology-Oncology-BMT, Department of Pediatrics, Emory University School of Medicine, Atlanta, GA 30322, United States; Aflac Cancer and Blood Disorders Center, Children’s Healthcare of Atlanta, Atlanta, GA 30322, United States; Division of Pediatric Hematology-Oncology-BMT, Department of Pediatrics, Emory University School of Medicine, Atlanta, GA 30322, United States; Aflac Cancer and Blood Disorders Center, Children’s Healthcare of Atlanta, Atlanta, GA 30322, United States; Division of Pediatric Hematology-Oncology, Yale School of Medicine, New Haven, CT 06520, United States; Division of Pediatric Hematology-Oncology-BMT, Department of Pediatrics, Emory University School of Medicine, Atlanta, GA 30322, United States; Aflac Cancer and Blood Disorders Center, Children’s Healthcare of Atlanta, Atlanta, GA 30322, United States; Division of Pediatric Hematology-Oncology, Yale School of Medicine, New Haven, CT 06520, United States

**Keywords:** sickle cell, pain, vaso-occlusive episode, transplant, high-impact chronic pain

## Abstract

**Introduction/Objective:**

Lack of a well-characterized phenotype of High-Impact Chronic pain (HICP), that is, chronic pain (CP) with substantial restriction of participation in work, social, or self-care activities remains a critical gap in identifying individuals with CP and SCD at-risk for poor pain outcomes.

**Methods:**

Retrospective study using the Electronic Health Record (EHR) at a large academic children’s hospital.

**Results:**

We report the clinical phenotype of 46 children with SCD diagnosed with HICP at time of consultation for Hematopoietic Cell Transplant (HCT). The mean age was 14.5 years (SD 3.9), 50% (*n* = 23) were female, 84.8% (*n* = 39) had HbSS genotype or similar, 30.4% (*n* = 14) had avascular necrosis, 84.8% (*n* = 39) were prescribed at least one disease modifying medication, and 41.3% (*n* = 19) were prescribed adjuvant analgesics. The cohort experienced a median of 6 (IQR 2, 9) and 8.50 (IQR 4.25, 15) episodes of healthcare utilization (HCU) for pain in 12 months and 24 months prior to the HCT consult, respectively, but about one-third did not experience frequent HCU (three or more episodes/year) for pain. In the 10 years leading up to the HCT consult, the incidence of HCU for pain year-over-year increased on an average by 15%. Clinical correlates of HICP from the EHR like prescription of adjuvant analgesics, cumulative doses of prescribed opioids, and diagnosis codes for CP were more likely to identify those who experienced frequent HCU for pain.

**Conclusion:**

HICP in SCD is associated with substantial morbidity. This study underscores the importance of screening for HICP in SCD.

## INTRODUCTION

Sickle cell disease (SCD), an inherited red blood cell disorder, is characterized by multi-system complications,^1^ poor health-related quality of life,^2^ and premature mortality.^3^^,^^4^ Chronic pain (CP), that is, pain on most days of the month for 6 months or more,^5^ is increasingly recognized as a major complication of SCD^6–8^ and is associated with substantial morbidity and poor outcomes.^9–11^ The current definition of CP^5^ in SCD incorporates frequency, that is, pain on most days and duration, that is, lasting ≥6 months, but this definition does not capture activity limitations or disability, or consider the heterogeneity in the impact of CP on an individual. The US National Pain Strategy report from the Interagency Pain Research Coordinating Committee^12^ called for separating those with CP who experience substantial restriction of participation in work, social, or self-care activities as High-Impact Chronic Pain (HICP), a severely impacted sub-group of individuals with CP. In the 2016 National Health Interview Survey, HICP was estimated to affect 8% of the U.S. population.^13^ Individuals with HICP were more likely to be older, female, achieve lower levels of education, live in rural areas, and live in poverty.^13–15^ Individuals with HICP also report more pain, pain interference, worse health including mental health and cognitive impairments, and higher healthcare use.^14^^,^^16^ While HICP has been examined in large epidemiological or observational,^13^^,^^14^^,^^17^^,^^18^ in clinical cohorts of individuals^19–21^ with CP conditions, and in a secondary analyses of the Pain in Sickle Cell Epidemiology Study^22^ in SCD, the clinical phenotype of HICP has not been characterized in SCD. This characterization is essential to identify individuals with CP and SCD who are most likely to experience poor pain outcomes and who may be referred for multi-disciplinary pain care and for consideration of novel therapies, including curative and transformative therapies.

In 2019, the disease severity criterion for inclusion in BMT CTN 1503 (NCT02766465), a clinical trial of Hematopoietic Cell Transplantation (HCT), a potentially curative treatment for SCD, were expanded to include HICP.^23–25^ This prompted the practice to incorporate screening for HICP as a marker of disease severity at time of HCT consultation as part of clinical care. This provided a unique opportunity for investigators to retrospectively characterize the clinical phenotype of children and adolescents with SCD who were identified as having HICP during outpatient consultation for HCT. In a crucial first step, we used Electronic Health Record (EHR) data sources to describe the clinical phenotype of HICP, including the longitudinal trends in pain episode frequency leading up to the time of diagnosis of HICP. We also proposed to compare the clinical phenotype of HICP by sex, and by the presence of high healthcare utilization for pain.

## METHODS

### Study design and objectives

We conducted a retrospective study of individuals with SCD diagnosed with HICP at the time of consultation for HCT in 2019-2020. Data were collected from the EHR at Children’s Healthcare of Atlanta, Atlanta, GA, a large multi-campus academic children’s hospital in a large metropolitan area in the south-eastern United States. The objective of this study was to use EHR-data sources to (1) describe the demographic and clinical correlates of HICP, including frequency of International Classification of Diseases (ICD)-10 CP diagnoses codes; in the full cohort, by sex, and by history of frequent healthcare utilization for pain; (2) evaluate for longitudinal trends in pain episode frequency for up to 10 years leading up to the time of diagnosis of HICP at consultation for HCT; in the full cohort, and by age and sex; and (3) explore for latent classes of individuals based on longitudinal trends in pain episode frequency leading up to the to the time of diagnosis of HICP.

### Inclusion/exclusion criteria

Individuals with SCD (any genotype) <22 years of age who attended a consultation for HCT and were diagnosed with HICP as described above during the consultation were included. To assess for inclusion, we reviewed the consult visit note to ascertain for physician documentation for the presence of HICP. Three items drawn and adapted from the National Pain Strategy (NPS) Pilot Test were used^16^; one item assessed for the presence of CP, and among those with CP, two assessed for the presence of HICP.^16^ The presence of CP was ascertained by a “yes” response to the question of whether an individual had SCD pain on “at least half the days” per month for the past 6 months. This definition of CP was similar to, but not identical to, the AAPT frequency criterion for the diagnosis of CP in SCD (ie, pain on “most days” for the past 6 months^5^). Among those with CP, the presence of HICP was determined according to the NPS pilot test items used for classification of chronic pain impact. An individual was determined to have HICP based on a response of “usually” or “always” when asked how often pain limited life or work activities, including household chores; or a response of “severe” interference when asked how much pain interfered with life activities.^16^ A structured template was typically used for clinical documentation. If responses to the individual items were not documented in the structured template, individuals were only included in this cohort if there was physician documentation in the consult note that they had chronic pain consistent with the diagnosis of HICP. If an individual had multiple consultations within our timeframe, the first outpatient consultation in the study period was used as the referent consult visit, unless an assessment for HICP occurred at a subsequent visit and not the first visit, in which case the visit where assessment for HICP was done was used as the referent consult visit.

We excluded individuals who did not meet inclusion criteria, or who were not screened for HICP, or if there was absence of documentation regarding diagnosis of HICP.

### Clinical phenotype characteristics

Clinical phenotype characteristics were abstracted from the EHR by manual review by the study investigators. HCU for pain included acute care visit (Emergency Department or Day-Hospital) or hospitalization for pain for up to 10 years from the referent consult visit. Records from each acute care visit or hospitalization were reviewed by study investigators to ensure that it was consistent with a visit for acute sickle cell pain,^3^ and elective admissions (eg, for surgeries or procedures) were excluded. To examine pain episode frequency near the time of consultation for HCT, we also calculated total HCU for pain (sum of all emergency department visits, day-hospital visits, and inpatient hospitalizations for pain) in the 12-month and 24-month periods up to the referent consult visit. Individuals were considered to have frequent HCU for pain if they had ≥3 episodes of HCU for pain in the 12 months up to the referent consult visit, a threshold associated with worse outcomes in SCD.^26^ We reviewed available reports of radiological studies as well as clinical notes to identify those consistent with a diagnosis of avascular necrosis (AVN). We collected data on prescribed medications including disease-modifying therapies, opioid, non-opioid, and adjuvant analgesics including neuropathic pain medications (NeP) and muscle relaxants in the 12 months prior to the referent consult visit. We also noted use of chronic transfusion therapy within 3 months of the referent consult visit. To calculate cumulative doses of prescribed oral opioids, we abstracted data from outpatient prescriptions recorded in the EHR in the 12 months prior to the referent consult visit, specifically opioid type, strength per unit of opioid, and number of units (or amount of liquid) of opioid prescribed per prescription.^27^ We then converted prescribed oral opioids to Morphine Milligram Equivalent (MME) doses using the conversion tables from the 2022 Centers for Disease Control (CDC) Clinical Practice Guideline for prescribing opioids for pain^28^ as follows: Hydrocodone: 1 MME, Oxycodone: 1.5 MME, Hydromorphone: 5 MME, Morphine: 1 MME, and Methadone: 4.7 MME. The total MME doses of opioids were then calculated per day and per kilogram body weight/day. Laboratory values were abstracted from the consult visit date, and if not available, then they were abstracted from the most-recent outpatient visit in the 6 months prior to the consult visit. We obtained all visit-level diagnosis codes up to 2 years from the referent clinic visit. We drew from ICD-10 codes used to study diverse CP conditions from Mayhew et al.^29^ and selected all codes (G89.0, G89.29, G89.4, F.45.42) from the diagnosis sub-cluster of general pain, belonging to a larger diagnosis cluster of other painful conditions.

### Statistical analyses

Primary statistical analysis was performed in CRAN R v.4.0.2 (Vienna, Austria) and SAS v.9.4 (Cary, NC). We used descriptive statistics to characterize demographic, clinical, and pain-related variables. Hypothesis testing was done using Chi^2^ test with Yates continuity correction/Fisher exact for categorical variables, and *t*-test with equal variance assumption or non-parametric Kruskal Wallis test for continuous variables. To describe the long-term trajectories of those with CP, we utilized mixed effects negative binomial regression models to estimate the longitudinal frequency of HCU for pain/year leading up to HCT consultation, expressed as an Incidence Rate Ratio (IRR) with 95% confidence intervals (CI). Considered fixed effects included time (as number of years prior to consult), patient sex, and the time by sex interaction. Random effects were subject-specific intercepts. As an exploratory analysis, we extended our negative binomial regression procedures to incorporate latent class growth analysis (LCGA) methods, to investigate for latent sub-classes of individuals with respect to frequency of HCU for pain/year leading up to HCT consultation. The only regression input for LCGA was frequency of HCU for pain/year and up to three latent classes were considered. The best model was determined by model fit statistics (ie, log-likelihood, AIC, and adjusted-BIC), entropy, the Lo–Mendell–Rubin likelihood ratio test, and clinical relevance. LCGA analyses were performed with maximum likelihood estimation with robust standard errors in Mplus v.8.6 (Los Angeles, CA).

This study was approved by the Institutional Review Board at Children’s Healthcare of Atlanta.

## RESULTS

Forty-six individuals met criteria for HICP. Of those 46, 42 had either a response of “usually” or “always” when asked how often pain limited life or work activities, including household chores or a response of “moderate” or “severe” interference when asked how much pain interfered with life activities documented in the EHR. For the remaining four individuals, the response to items on pain frequency and persistence were not documented, but physician documentation noted that they had HICP. Item-level responses were not recorded for all individuals for every item. Of the individuals for whom item-level responses were available, 37 (of 42 available responses, 88%) reported “severe interference” in response to how much pain interfered with life or work activities, 39 (of 40 available responses, 97.5%) reported “usually” or “always” in response to how often pain interfered with life or work activities, and 34 reported both “severe interference” in response to how much pain interfered with life activities and “usually” or “always” in response to item asking about frequency of pain interference. All but one who reported severe pain interference also reported frequent pain interference.

### Clinical phenotype

Clinical phenotype and correlates are reported in Table 1. The mean age was 14.5 years (SD 3.9), 50% (*n* = 23) were female, and 84.8% (*n* = 39) had HbSS genotype or similarly severe genotype. Overall, individuals with HICP experienced high HCU for pain, with a median of 6 (Interquartile Range, IQR 2, 9) episodes in the 12 months prior to consult, and 65.2% (*n* = 30) had ≥3 visits for SCD-related pain. The median number of HCU for pain in 24 months (2 years) prior to the consult visit was 8.50 (IQR 4.25, 15), and 65.2% (*n* = 30) had ≥6 visits for SCD-related pain. Fourteen (30.4%) had a known diagnosis of AVN. Most (84.8%, *n* = 39) were prescribed at least one disease-modifying medication in the 12 months preceding the consult. Thirteen percent (*n* = 6) received chronic transfusion therapy within the 3 months preceding the consult. Almost all (93.5%, *n *= 43) were prescribed one or more short-acting opioids, and very few (6.5%, *n* = 3) were prescribed one or more long-acting opioids within the 12 months prior to the consult visit. The median cumulative doses described in morphine milligram equivalent/day (MME/day) and morphine milligram equivalent//kilogram/day (MME/kg/day) were overall low. Adjuvant analgesics, which included neuropathic pain medications or muscle relaxants, were prescribed in 41.3% (*n* = 19) participants.

**Table 1.** Clinical phenotype characteristics of HICP.

Characteristic (*n* = 46)	
Age, mean (SD)	14.5 (3.9)
Sex, *n* (%)	
Female	23 (50.0)
Male	23 (50.0)
Body mass index, *n* (%)	
Underweight (<18.5 or <5th percentile)	3 (6.5)
Healthy weight (18.5 to <25 or 5th to <85th percentile)	34 (73.9)
Overweight or obese (25 and higher or 85th percentile or higher)	9 (19.6)
Genotype	
HbSS/HbSβ^0^ thalassemia/HbS-OArab	39 (84.8)
HbSC/HbSβ^+^ thalassemia	7 (15.2)
Healthcare utilization for pain	
In year prior to consult, median [IQR]	6.00 [2.00, 9.00]
Proportion with ≥2 episodes in year prior to consult	36 (78.3)
Proportion with ≥3 episodes in year prior to consult	30 (65.2)
Proportion with ≥4 episodes in year prior to consult	29 (63.0)
Proportion with ≥5 episodes in year prior to consult	27 (58.7)
In 2 years prior to consult, median [IQR]	8.50 [4.25, 15.00]
Proportion with ≥6 episodes in 2 years prior to consult	30 (65.2)
Presence of AVN, *n* (%)	14 (30.4)
Disease-modifying medications in year prior to consult, *n* (%)	39 (84.8)
Hydroxyurea	37 (80.4)
L-glutamine	11 (23.9)
Chronic transfusion therapy in 3 months prior to consult	6 (13.0)
Short-acting oral opioid (hydrocodone/oxycodone/hydromorphone/morphine immediate release/tramadol), *n* (%)	43 (93.5)
Long-acting oral opioid (morphine extended release/methadone), *n* (%)	3 (6.5)
Morphine milligram equivalents prescribed in past year/day^a^	
Total, median [IQR]	3.54 [1.36, 8.71]
Per kilogram body weight, median [IQR]	0.08 [0.03, 0.16]
NSAID, *n* (%)	43 (93.5)
Ibuprofen/naproxen, *n* (%)	42 (91.3)
Celecoxib/meloxicam, *n* (%)	6 (13)
All adjuvant analgesics (neuropathic pain medications/muscle relaxants), *n* (%)	19 (41.3)
Neuropathic pain medications, *n* (%)	14 (30.4)
Clonidine, *n* (%)	6 (13.0)
Gabapentin/pregabalin, *n* (%)	10 (21.7)
Amitriptyline/nortriptyline, *n* (%)	2 (4.3)
Muscle relaxants (cyclobenzaprine/methocarbamol/tizanidine), *n* (%)	13 (28.3)
Laboratory values^b^	
Hemoglobin (g/dL), mean (SD)	9.4 (1.1)
Mean corpuscular volume (fL), mean (SD)	94.1(13.6)
White blood cell count (K/mcL), mean (SD)	9.5 (3.0)
Platelet count (K/mcL), mean (SD)	392.9 (173.1)
Presence of ICD-10 diagnosis code for chronic pain^c^, *n* (%)	
Past year	15 (37.5)
Past 2 years	17 (41.5)

a *n* = 40.

b *n* = 39.

c ICD codes available for *n* = 40 for past year and *n* = 41 for past 2 years.

Of the individuals for whom visit level ICD10 diagnoses codes were available, we found that 17 individuals (41.5% of 41 individuals) had at least one encounter with a diagnosis code for CP in the 2 years prior to the consult for HCT, and 15 (37.5% of 40 individuals) had at least one encounter with a diagnosis code for CP in the 1 year prior to the consult for HCT.

### Clinical phenotype differences by sex or frequent HCU for pain

We did not find any significant differences in clinical phenotype correlates reported in this study by sex. We found that individuals with frequent HCU for pain in the past year were more likely to be prescribed higher cumulative doses of opioids (*P* < .001), and adjuvant analgesics (*P* = .010), including NeP medications (*P* ≤.001). Frequent HCU for pain was associated with a concurrent ICD-10 diagnosis code for CP in the 12 months (*P* = .001) and 24 months (*P* = .004) prior to the consult. Results are reported in Table 2.

**Table 2.** Clinical phenotype characteristics of HICP compared by frequency of healthcare utilization for pain in year (12 months) prior to consult for HCT.

	Healthcare utilization for pain in year prior to consult		
Characteristic (*n* = 46)	<3 in past year	3 or more	*P*
***n***	16	30	
Age, mean (SD)	13.8 (4.5)	14.9 (3.6)	0.334
Sex, *n* (%)			0.353
Female	10 (62.5)	13 (43.3)	
Male	6 (37.5)	17 (56.7)	
Body mass index *n* (%)			
Underweight (<18.5 or <5th percentile)	2 (12.5)	1 (3.3)	0.274
Healthy weight (18.5 to <25 or 5th to <85th percentile)	12 (75)	22 (73.3)	1
Overweight or obese (25 and higher or 85th percentile or higher)	2 (12.5)	7 (23.3)	0.463
Genotype, *n* (%)			0.681
HbSS/HbSβ^0^ thalassemia/HbS-OArab	13 (81.2)	26 (86.7)	
HbSC/HbSβ^+^ thalassemia	3 (18.8)	4 (13.3)	
Healthcare utilization for pain			
In year prior to consult, median [IQR]	1.00 [1.00, 2.00]	7.00 [6.00, 11.50]	**<0.001**
In 2 years prior to consult, median [IQR]	2.50 [1.75, 5.00]	12.50 [8.25, 17.75]	**<0.001**
Presence of AVN, *n* (%)	6 (37.5)	8 (26.7)	0.512
Disease-modifying therapy in year prior to consult, *n* (%)	14 (87.5)	25 (83.3)	1
Hydroxyurea	14 (87.5)	23 (76.7)	0.463
L-glutamine	2 (12.5)	9 (30.0)	0.282
Chronic transfusion therapy in 3 months prior to consult	1 (6.2)	5 (16.7)	0.649
Short-acting oral opioid (hydrocodone/oxycodone/hydromorphone/morphine immediate release/tramadol), *n* (%)	15 (93.8)	28 (93.3)	1
Long-acting oral opioid (morphine extended release/methadone), *n* (%)	0 (0.0)	3 (10.0)	0.542
Morphine milligram equivalents prescribed in past year/day^a^			
Total, median [IQR]	1.66 [0.59, 1.78]	5.56 [3.47, 12.54]	**<0.001**
Per kilogram body weight, median [IQR]	0.03 [0.02, 0.05]	0.11 [0.08, 0.22]	**<0.001**
NSAID, *n* (%)	14 (87.5)	29 (96.7)	0.274
Ibuprofen/naproxen, *n* (%)	14 (87.5)	28 (93.3)	0.602
Celecoxib/meloxicam, *n* (%)	0 (0.0)	6 (20.0)	0.078
All adjuvant analgesics (neuropathic pain medications/muscle relaxants), *n* (%)	2 (12.5)	17 (56.7)	**0.010**
Neuropathic pain medications, *n* (%)	0 (0.0)	14 (46.7)	**<0.001**
Clonidine, *n* (%)	0 (0.0)	6 (20.0)	0.078
Gabapentin/pregabalin, *n* (%)	0 (0.0)	10 (33.3)	**0.009**
Amitriptyline/nortriptyline, *n* (%)	0 (0.0)	2 (6.7)	0.536
Muscle relaxants (cyclobenzaprine/methocarbamol/tizanidine), *n* (%)	2 (12.5)	11 (36.7)	0.101
Laboratory values^b^			
Hemoglobin (g/dL), mean (SD)	9 (1.1)	9.6 (1.1)	0.082
Mean corpuscular volume (fL), mean (SD)	94.8 (13.5)	93.7 (13.9)	0.817
White blood cell count (K/mcL), mean (SD)	9.9 (3.4)	9.3 (2.8)	0.599
Platelet count (K/mcL), mean (SD)	444.1 (127.5)	367.3 (188.9)	0.195
Presence of ICD-10 diagnosis code for chronic pain,^c^ *n* (%)			
Past year	0 (0.0)	15 (57.7)	**0.001**
Past 2 years	1 (7.1)	16 (59.3)	**0.004**

a *n* = 40.

b *n* = 39.

c ICD codes available for *n* = 40 for past year and *n* = 41 for past 2 years. p<0.05 is highlighted in bold.

We found similar results when we compared those who had an ICD-10 diagnosis code for CP in the past year with those who did not, and results are reported in Table S1.

### Evolution of pain episode frequency over time

**Figure 1.**
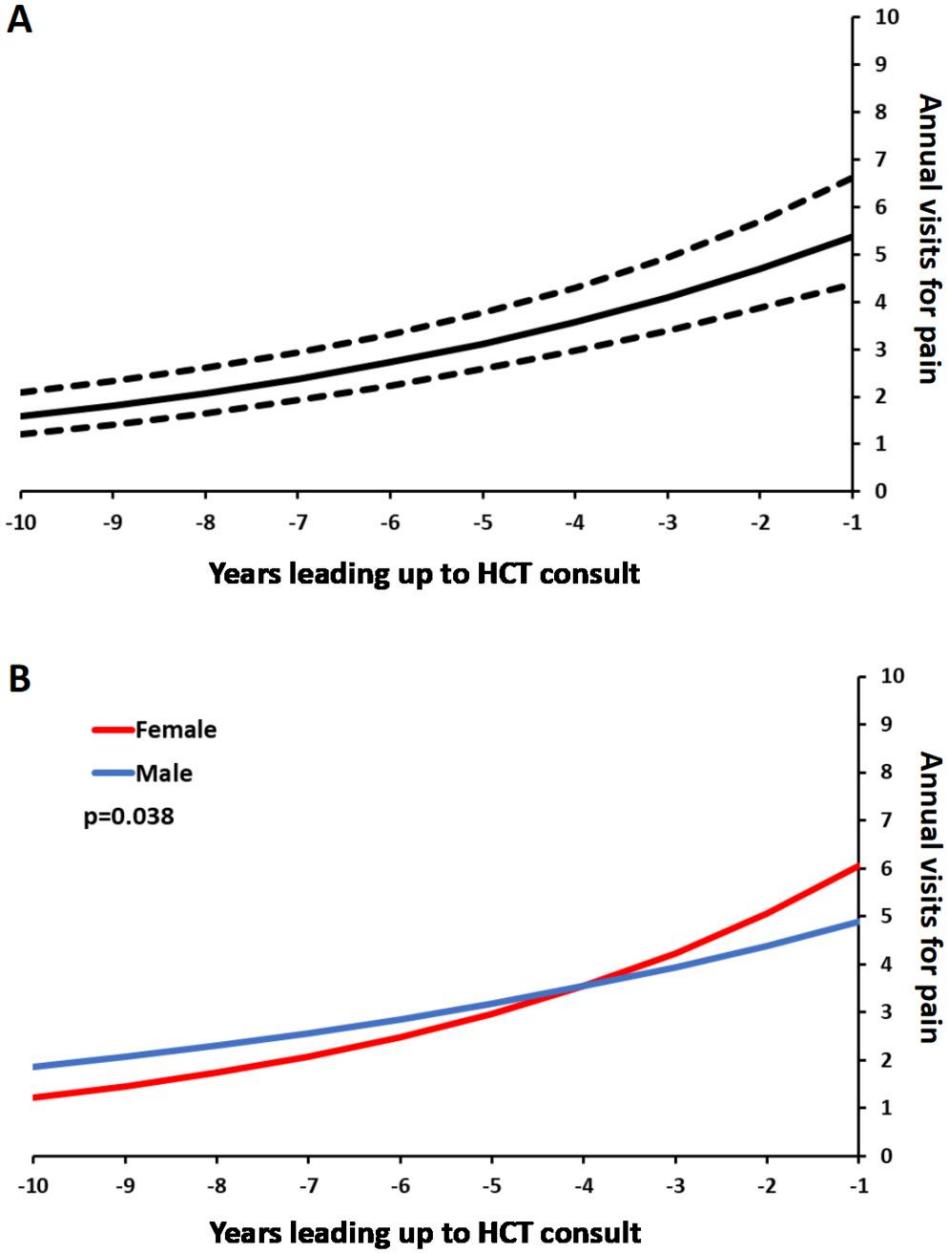
Mixed effects negative binomial growth curves with 95% CI up to 10 years prior to consultation for HCT(*n *= 46). (A) Model estimated curves with *y*-axis truncated to max of 10 episodes of healthcare utilization for pain/year. (B) Model estimated curves by sex and with *y*-axis truncated to max of 10 episodes of healthcare utilization for pain/year.

In the 10 years prior to the referent HCT consult visit, we observed inter-individual variability in the frequency of HCU, and an overall increase in the frequency of HCU for pain closer to the consult visit. Expressed as an incidence rate ratio (IRR), we found that the incidence of HCU year-over-year increased on an average by 15% (Table 3 and Figure 1). Considering this trend by patient sex, we found that the incidence rate of healthcare visits year over year leading up to the HCT consult increased by 20% for females, and 11% for males, a difference which was statistically significant (*P* = .038, Table 3 and Figure 1). Multiple sensitivity models were evaluated to confirm the robustness of the primary model. The direction and approximate strength of relationships from the primary analyses were consistent in the sensitivity models as reported in Table 3. Of note, the statistically significant difference in trends between sexes in the primary analysis did not hold for all sensitivities, as reported in Table 3.

**Table 3.** Incidence rate ratios (IRR) and 95% CI using mixed effects negative binomial regression with healthcare utilization/year as a count outcome. Results from primary and sensitivity analyses are shown.

Sample	IRR (95% CI)	*P* value
***Primary model—all data up to 10 years prior to consult***		
Overall (*n *= 46)	1.15 (1.11, 1.19)	**<0.001**
Birth sex (*n *= 46)		
Female	1.20 (1.14, 1.26)	**0.038**
Male	1.11 (1.07, 1.16)	
***Sensitivity model 1—all data up to 5-years prior to consult***		
Overall (*n *= 46)	1.16 (1.08, 1.25)	**<0.001**
Birth sex (*n *= 46)		
Female	1.21 (1.09, 1.34)	0.252
Male	1.11 (1.01, 1.23)	
***Sensitivity model 2—all data up to 10 years prior to consult, with visits >20/year truncated to 20/year* ^a^**		
Overall (*n *= 46)	1.14 (1.11, 1.18)	**<0.001**
Birth sex (*n *= 46)		
Female	1.18 (1.13, 1.24)	0.066
Male	1.11 (1.07, 1.16)	
***Sensitivity model 3—all data up to 10 years prior to consult, excluding individuals with >20 visits in any year***		
Overall (*n *= 44)	1.13 (1.09, 1.17)	**<0.001**
Birth sex (*n *= 44)		
Female	1.16 (1.10, 1.23)	0.226
Male	1.11 (1.07, 1.16)	
***Sensitivity model 4—all data up to 10 years prior to consult, limited to age >12 years at time of consult visit***		
Overall (*n *= 36)	1.14 (1.10, 1.18)	**<0.001**
Birth sex (*n *= 36)		
Female	1.20 (1.13, 1.27)	**0.032**
Male	1.11 (1.06, 1.16)	
***Sensitivity model 5—all data up to 10 years prior to consult, limited to age >12 years at time of consult visit, and excluding individuals with >20 visits in any year***		
Overall (*n *= 34)	1.12 (1.09, 1.16)	**<0.001**
Birth sex (*n *= 34)		
Female	1.16 (1.09, 1.23)	0.238
Male	1.11 (1.06, 1.15)	

a Yearly visits >20 were truncated to 20 (a total of 4 years of data in two patients). p<0.05 is highlighted in bold.

### An exploratory analysis of sub-classes of longitudinal trajectories among individuals with HICP

**Figure 2.**
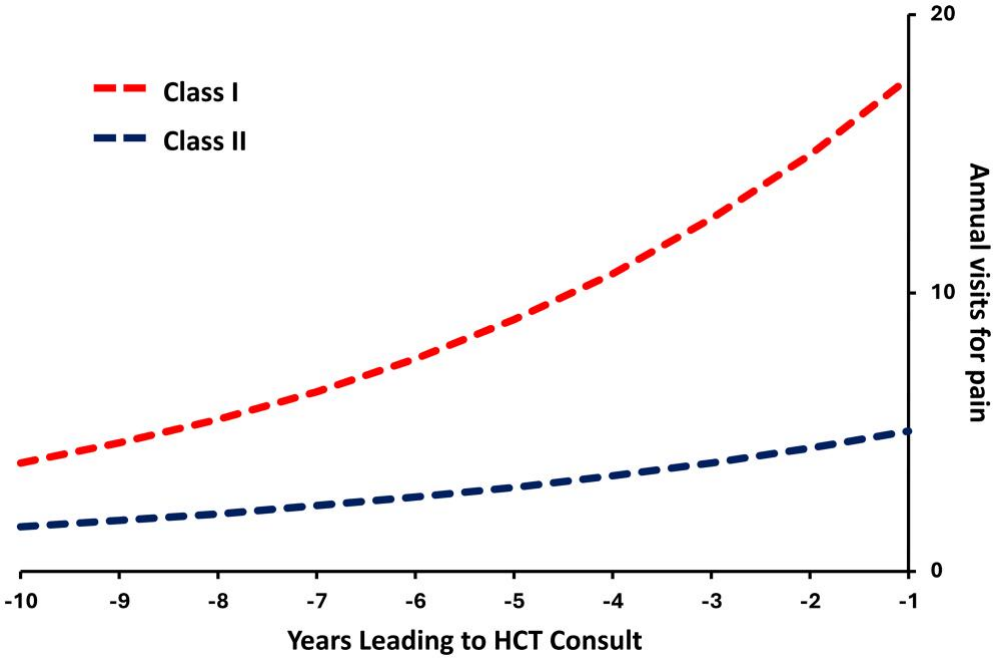
Latent-class growth model classifications and model-estimated curves of healthcare utilization for pain/year.

LCGA pointed to two distinct classes of patients (Table S2 and Figure 2), with five individuals in Class I and 41 individuals in Class II. Individuals in Class I had higher median HCU for pain as compared to those in Class II. This was observed for the 12 months [12 (IQR 9, 13) compared to 5 (IQR 2, 7), *P* = .010] and 24 months [29 (IQR 19, 31) compared to 7 (IQR 4, 12), *P* = .002] preceding the HCT consult visit. Individuals in Class I had been prescribed higher cumulative doses of opioids in the year preceding the HCT consult, with a median MME/kg/day of 0.32 (IQR 0.28, 0.33) in Class I compared to 0.07 (IQR 0.03, 0.11), *P* = .001 in Class II. Individuals in Class I were more likely to be prescribed Celecoxib or Meloxicam compared to those in Class 2 (60% compared to 7.3%, *P* = .012). Lastly, all individuals in Class I had an ICD-10 diagnosis for CP in the 1 year (compared to 28.6% of those in Class II, *P* = .005) and 2 years (compared to 33.3% of those in Class II, *P* = .008) leading up to the consult for HCT. There were no other significant differences between individuals who were in Class I or II for the remaining variables reported in Table 1.

## DISCUSSION

The results of this study represent the first description of the clinical phenotype of HICP using EHR data. Overall, the study confirmed the high burden of disease morbidity experienced by children with SCD who attend a consultation for HCT and have HICP.

Most individuals in this cohort experienced frequent HCU for pain, more frequent than previous reports in SCD.^30^ A high pain burden was also reported in individuals with a HICP-like phenotype in the Pain in Sickle Cell Epidemiology Study.^22^ For the overall cohort, there was significant year-over-year increase in frequency of HCU for pain leading up to the consult for HCT. While these trends suggest increasing pain frequency over time, it is possible that the trends in HCU are confounded by increase in pain rates with age^31^ or by the timing of referral for consultation for HCT. An increase in frequency of HCU may have prompted a referral for HCT consultation by their healthcare provider,^32^ or may have prompted patients and families to request a HCT consultation.^33^ We found that females appeared to have a higher year-over-year increase in HCU for pain over time, as compared to the males, but this finding was not consistently statistically significant in sensitivity analyses. While higher prevalence of HICP has been reported among women in the NHIS,^13^ in our limited sample we did not find any differences in distribution or observed clinical variables by sex, but the sample size of this study precludes any conclusion regarding the role of sex in HICP in SCD. Exploratory analyses suggest there may be variability in pain frequency trajectories among individuals with HICP, though the small sample size precludes definite conclusions. Trends in pain frequency and the possibility of sub-phenotypes of growth trajectories among individuals with HICP and the role of sex in HICP in SCD should be examined in future studies.

About one-third of individuals in this cohort had AVN, consistent with previous observations that AVN occurs frequently in CP,^5^^,^^34^^,^^35^ is associated with frequent HCU^36^^,^^37^ and 30-day readmissions in SCD, and is often reported in individuals with SCD and CP undergoing HCT.^38^ Almost all individuals were prescribed one or more disease-modifying therapies, which may reflect more severe disease.^39^ This may also reflect a referral bias, given that a referral for HCT may be considered based on response (or lack thereof) to current therapies,^32^ or consultation may only be sought by families as a “last resort,”^33^ after exhausting other treatment options. Lastly, about 40% of individuals were prescribed one or more adjuvant analgesics, including 30% who were prescribed NeP medications, which has been previously reported amongst those with SCD and CP.^35^^,^^40^ NeP is believed to be a contributor to pain and CP in SCD^41–44^ and prescription of adjuvant analgesics is consistent with the recent American Society of Hematology guidelines for the management of CP in SCD.^45^ These findings should be confirmed in future studies.

This study also provided some insights into using surrogate markers from the EHR to identify people with CP in SCD. Importantly, not all individuals with HICP experienced frequent HCU for pain, and screening for HICP identifies a subgroup who experiences CP and disability but does not experience high healthcare utilization for pain. Less than half of the individuals in this cohort had a diagnoses code for CP, and the association of diagnoses codes for CP with frequent HCU for pain suggested that those with frequent HCU for pain were more likely to be identified as having HICP. Similarly, the use of NeP medications or adjuvant analgesics as surrogates for HICP may pose a challenge as the prescription of these medications was associated with frequent HCU for pain. The prescription of adjuvant analgesics in this cohort may also be confounded by the frequency of contact with the healthcare system for pain. The overall daily outpatient prescribed MME in children with HICP and SCD were lower than that previously reported in adults with SCD and chronic pain.^46^^,^^47^ Thus, daily outpatient opioid MME may not be an accurate surrogate of HICP, particularly in the pediatric population. The results of this study support screening for HICP using established criteria,^5^^,^^48^ as use of EHR surrogate markers alone to identify HICP is likely to miss many individuals with HICP. Screening for HICP would allow clinicians to identify those with CP at high risk for poor outcomes for referral for multi-disciplinary CP care, and for consideration of disease modifying, curative, or transformative therapies. Stratification based on impact of CP also allows for reduction in disability and improved function to be investigated as treatment endpoints in intervention studies. Lastly, screening for CP and HICP may allow for early identification of individuals with CP without HICP for interventions, and future research should examine if early intervention may prevent transition to HICP.

Our study has several limitations. The individuals in this study with SCD and HICP were identified at the time of a consultation for HCT in a limited timeframe between 2019 and 2020. Given the institutional practice of accepting referrals to discuss HCT regardless of disease severity or availability of a donor, the individuals attending consultation for HCT may not have been representative of individuals attending consultation for HCT in institutions that may use different considerations for referrals for HCT. Additionally, not all patients with SCD and CP desire a consult for HCT, and system- and patient-related factors may not allow a patient who is referred to attend a consult for HCT.^32^ Patients with SCD who completed consultation for HCT but who may not have been assessed for HICP were excluded, as were those who were not referred for consultation for HCT. Since this study focused on children attending an HCT consultation, it is possible that the findings were biased toward increased symptom and disease severity. Clinical correlates of HICP identified in this study, such as prescription of adjuvant analgesics or specific NSAIDs, may reflect institutional practice and may not be representative of medications used by people with SCD and HICP. These factors may limit generalizability of the findings and may not be representative of all individuals with SCD and HICP. Lastly, individuals with SCD were assessed for CP using items from the NPS pilot test,^16^ which used the term “at least half the days,” rather than the AAPT criteria^5^ used “most days” as a frequency criterion to define CP. There may be differences in response to item wording between these two definitions when assessing for the presence of CP in SCD. The items from the NPS pilot test^16^ that were used to assess HICP were not formally validated in children, or in SCD, but were administered in a clinical care setting, which provided the opportunity for the consulting physician to ensure the item was understood, and to clarify patient responses. The items in the NPS pilot test^16^ have since been modified, and the Graded Chronic Pain Scale-Revised (GCPS-R) is currently used to measure CP impact and identify individuals with HICP^48^; these factors should be considered when comparing results of this study with those of future studies.

The findings in this study were based on data available in the EHR, so it is possible that some clinical characteristics or episodes of HCU for pain outside our institution were not captured. Similarly, only visit-level diagnoses codes from the EHR were available. Given the nature of EHR data, we were unable to confirm adherence to medications and were limited to medications prescribed in the EHR. Additionally, only outpatient opioid prescriptions in the EHR were used to calculate daily MME, and opioids prescribed at other sites or in the hospital during emergency room, day hospital, or inpatient stays were not accounted for, which underestimated the cumulative doses of opioids prescribed, especially for those individuals that experienced frequent or prolonged hospitalizations. Given the nature of EHR data, we could not collect data on psychological co-morbidities associated with HICP. Lastly, the results of this study are from a single large institution. Future multi-institutional studies should confirm these findings. Such studies should also prospectively study social determinants of health, as well as predictors, outcomes of HICP in SCD, and the longitudinal outcomes of HICP in SCD.

## CONCLUSIONS

We conclude that in SCD, the clinical phenotype of HICP is associated with substantial morbidity. The results of this study suggest formal screening to identify individuals with HICP as clinical correlates of HICP from the EHR are likely to identify those who experience frequent HCU for pain. HICP in SCD should be studied in prospective studies to guide risk-stratification of CP for clinical care and research.

## Supplementary Material

yoaf028_Supplementary_Data
