# Supplementary material for: Clinical phenotype of high-impact chronic pain in sickle cell disease at consultation for hematopoietic cell transplant
Source: J Sick Cell Dis. 2025 Aug 21;2(1):yoaf028. doi: 10.1093/jscdis/yoaf028 (PMC12476911; doi:10.1093/jscdis/yoaf028)
Supplement: yoaf028_Supplementary_Data [file yoaf028_supplementary_data.docx]

**SUPPLEMENTARY MATERIAL:**

**TITLE:** Clinical phenotype of high-impact chronic pain in sickle cell disease at consultation for hematopoietic cell transplant

**AUTHORS:** Serena Huang, BS^1^, Scott Gillespie MS, MSPH^2^, Eric Chou, MSPH^4^, Katie Liu, MSPH^2^, Ashna Jagtiani, MBBS, MPH^3,4^, ^#^Lakshmanan Krishnamurti, MD^3,4,5^, and ^#^Nitya Bakshi, MBBS, MS^3,4,5^

**AFFILIATIONS:**

1 Emory University School of Medicine, Atlanta, GA

2 Pediatrics Biostatistics Core, Department of Pediatrics, Emory University, Atlanta, GA

3 Aflac Cancer and Blood Disorders Center, Children’s Healthcare of Atlanta, Atlanta, GA

4 Division of Pediatric Hematology-Oncology-BMT, Emory University School of Medicine, Atlanta, GA.

5 Division of Pediatric Hematology-Oncology, Yale School of Medicine, New Haven, CT

#Co-senior author

**CORRESPONDING AUTHOR:** Nitya Bakshi, MBBS, MS

Email: nitya.bakshi@yale.edu

**Supplemental Tables:**

**Supplemental Table 1: Clinical phenotype characteristics of HICP compared by presence of ICD code for chronic pain in year prior to consult for HCT.**

| **Characteristic (n=40)** | **Absent** | **Present** | *p* |
| --- | --- | --- | --- |
| **n** | 25 | 15 |  |
| Age, Mean (SD) | 13.7 (4.0) | 15.3 (1.9) | 0.171 |
| Sex, n (%) |  |  |  |
| Female | 14 (56.0) | 8 (53.3) | 1 |
| Male | 11 (44.0) | 7 (46.7) |  |
| Body Mass Index |  |  |  |
| Underweight (<18.5 or <5th percentile), n (%) ^#^ | 3 (12.0) | 0 (0.0) | 0.279 |
| Healthy weight (18.5 to <25 or 5th to <85th percentile) | 21 (84.0) | 10 (66.7) | 0.256 |
| Overweight or Obese (25 and higher or 85th percentile or higher) | 1 (4.0) | 5 (33.3) | **0.021** |
| Genotype |  |  | 0.345 |
| HbSS/HbSβ^0^ thalassemia/HbS-OArab | 23 (92.0) | 12 (80.0) |  |
| HbSC/HbSβ^+^ thalassemia | 2 (8) | 3 (20) |  |
| Healthcare utilization for pain |  |  |  |
| In year prior to consult, Median [IQR] | 2.00 [1.00, 6.00] | 12.00 [8.00, 15.00] | **<0.001** |
| In 2 years prior to consult, Median [IQR] | 5.00 [2.00, 9.00] | 17.00 [13.50, 28.00] | **<0.001** |
| Presence of AVN, n (%) | 8 (32.0) | 4 (26.7) | 1 |
| Disease-modifying therapy in year prior to consult, n (%) | 20 (80.0) | 13 (86.7) | 0.691 |
| Hydroxyurea | 19 (76.0) | 13 (86.7) | 0.686 |
| L-glutamine | 4 (16.0) | 6 (40.0) | 0.135 |
| Chronic Transfusion Therapy in 3 months prior to consult | 2 (8.0) | 1 (6.7) | 1 |
| Short-acting oral opioid (Hydrocodone/Oxycodone/Hydromorphone/Morphine Immediate Release/Tramadol), n (%) | 25 (100.0) | 15 (100.0) | NA |
| Long-acting oral opioid (Morphine Extended Release/Methadone), n (%) | 0 (0.0) | 3 (20.0) | **0.046** |
| Morphine Milligram Equivalents prescribed in past year/day |  |  |  |
| Total, Median [IQR] | 1.77 [1.13, 3.39] | 11.05 [7.52, 20.51] | **<0.001** |
| Per kilogram body weight, Median [IQR] | 0.05 [0.03, 0.08] | 0.19 [0.11, 0.32] | **<0.001** |
| NSAID, n (%) | 24 (96.0) | 15 (100.0) | 1 |
| Ibuprofen/Naproxen, n (%) | 24 (96.0) | 14 (93.3) | 1 |
| Celecoxib/Meloxicam, n (%) | 0 (0.0) | 6 (40.0) | **0.001** |
| All adjuvant analgesics (Neuropathic pain medications or adjuvant analgesics), n (%) | 5 (20.0) | 13 (86.7) | **<0.001** |
| Neuropathic Pain Medications, n (%) | 3 (12.0) | 10 (66.7) | **0.001** |
| Clonidine, n (%) | 1 (4.0) | 5 (33.3) | **0.021** |
| Gabapentin/Pregabalin, n (%) | 1 (4.0) | 8 (53.3) | **<0.001** |
| Amitriptyline/Nortriptyline, n (%) | 1 (4.0) | 1 (6.7) | 1 |
| Muscle relaxants (Cyclobenzaprine/Methocarbamol/Tizanidine), n (%) | 2 (8.0) | 11 (73.3) | **<0.001** |
| Laboratory values^ |  |  |  |
| Hemoglobin (g/dL), Mean (SD) | 9.1 (1.0) | 9.9 (1.2) | **0.038** |
| Mean corpuscular volume (fL), Mean (SD) | 95.4 (12.0) | 92.1 (16.1) | 0.469 |
| White blood cell count (K/mcL), Mean (SD) | 9.8 (3.1) | 9.0 (2.9) | 0.405 |
| Platelet count (K/mcL), Mean (SD) | 414.8 (162.5) | 357.8 (189.2) | 0.323 |

^n=39

**Supplemental Table 2:** Latent class growth model fit statistics.

| **LCGA Solution** | **Log-Likelihood** | **AIC** | **Adjusted-BIC** | **Class. Quality**  **(Entropy)** | **Test vs. k-1 latent classes^1^** | **Class Sizes** |
| --- | --- | --- | --- | --- | --- | --- |
| 1 Class | -678.06 | 1362.13 | 1358.21 | -- | -- | 46 |
| **2 Classes** | **-644.90** | **1301.80** | **1293.96** | **0.950** | **0.004** | **5; 41** |
| 3 Classes | -637.69 | 1293.37 | 1281.61 | 0.721 | 0.279 | 5; 20; 21 |

^1^Based on Lo-Mendell-Rubin Adjusted LRT test
